# Predator-Induced Changes in Metabolism Cannot Explain the Growth/Predation Risk Tradeoff

**DOI:** 10.1371/journal.pone.0006160

**Published:** 2009-07-07

**Authors:** Ulrich K. Steiner, Josh Van Buskirk

**Affiliations:** 1 Zoological Institute, University of Zürich, Zürich, Switzerland; 2 Department of Biology, Stanford University, Stanford, California, United States of America; Northeastern University, United States of America

## Abstract

Defence against predators is usually accompanied by declining rates of growth or development. The classical growth/predation risk tradeoff assumes reduced activity as the cause of these declines. However, in many cases these costs cannot be explained by reduced foraging effort or enhanced allocation to defensive structures under predation risk. Here, we tested for a physiological origin of defence costs by measuring oxygen consumption in tadpoles (*Rana temporaria*) exposed to predation risk over short and long periods of time. The short term reaction was an increase in oxygen consumption, consistent with the “fight-or-flight” response observed in many organisms. The long term reaction showed the opposite pattern: tadpoles reduced oxygen consumption after three weeks exposure to predators, which would act to reduce the growth cost of predator defence. The results point to an instantaneous and reversible stress response to predation risk. This suggests that the tradeoff between avoiding predators and growing rapidly is not caused by changes in metabolic rate, and must be sought in other behavioural or physiological processes.

## Introduction

Organisms protect themselves against predators using a range of defence mechanisms, many of which are plastic and expressed only under predation risk [1]. In animals, most attention has been given to predator-induced changes in external morphology, behaviour, and life history, while underlying physiological responses remain little explored [2], [3]. The traditional view of induced behavioural defences is that predation risk leads to reduced activity of prey individuals, in turn reducing their encounter rate with, and detection by, predators [4]. However, reduced activity carries a cost, because less active animals spend less time searching for food and feeding. This leads to the so-called growth/predation risk tradeoff, which arises because the survival benefits of defence can only be obtained at the cost of reduced growth or development [3]–[5]. A similar argument applies to morphological defences, because resources invested in defensive morphologies are unavailable for growth [5], [6].

Recent work suggests that this traditional view is too simplistic; a more complex interplay between multiple interacting responses determines the effects of predators on traits such as growth, age, and size at metamorphosis. Although many studies confirm that predation risk causes reduced activity or increased refuge use [7], [8], and such reduced activity lowers predation rates [9], [10], these behavioural changes are often not directly associated with reduced growth or development [3], [11]–[15]. Consistent evidence of growth costs is also lacking for some well-studied morphological defences [1], [16], [17]. Two resolutions of this problem have been proposed. One is that decreased activity need not cause decreased food consumption, and therefore a growth or development cost is not an inevitable consequence of the behavioural response to predators [3], [13]. The second possibility is that, even if consumption is reduced in the presence of predators, compensatory physiological mechanisms can decouple growth rate from food consumption [3], [11], [15]. Physiological plasticity could occur in digestion and energy storage or in metabolism and respiration [15], [18], [19]. Data available so far suggest that digestive explanations cannot always explain the decoupling of behaviour and growth. For example, Steiner [13] discovered that amphibian larvae exposed to predators ingested the same amount of food with less feeding effort, and digested food more efficiently, compared to non-exposed individuals. Steiner therefore expected predator exposed tadpoles to grow or develop faster, but they did not. There is somewhat better support for the metabolic explanation, because brief exposure to predator cues causes increased ventilation, high heart beat rates, or high respiration rates in *Daphnia* [20], mussels [21], and fish [22], [23]. Thus, the growth/predation risk tradeoff may arise not only because prey reduce activity in dangerous situations, but also because predator-induced defences are associated with a costly increase in metabolic rate [24]–[26].

Our study focused on the metabolic explanation for the tradeoff between predator avoidance and growth or development. We tested whether the increase in oxygen consumption observed under short-term exposure to predators in other organisms occurs also in an amphibian larva, and whether that same metabolic response is maintained under more realistic conditions of chronic exposure over several weeks. Increased oxygen consumption – indicative of an increased metabolic rate – could explain growth and development costs of responding to predators despite no reduction in food consumption or digestion efficiency. Identifying physiological mechanisms that help shape the growth/predation risk tradeoff is important for understanding the costs and benefits of phenotypic plasticity and how they influence species distributions with respect to predators [2], [3], [27], [28].

## Results

Conditioned tadpoles (reared with predators) were smaller than naïve tadpoles on average (mass±SE: 466±21 mg versus 579±25 mg; F_1,19_ = 21.54, p<0.0002; based on 6 individuals per pool sampled at age 28 days). Mass after conditioning is a direct measure of growth rate, because sizes of randomly-assigned tadpoles did not differ at the onset of the experiment. This confirms many previous studies showing reduced growth of predator-exposed tadpoles [29].

**Figure 1:**
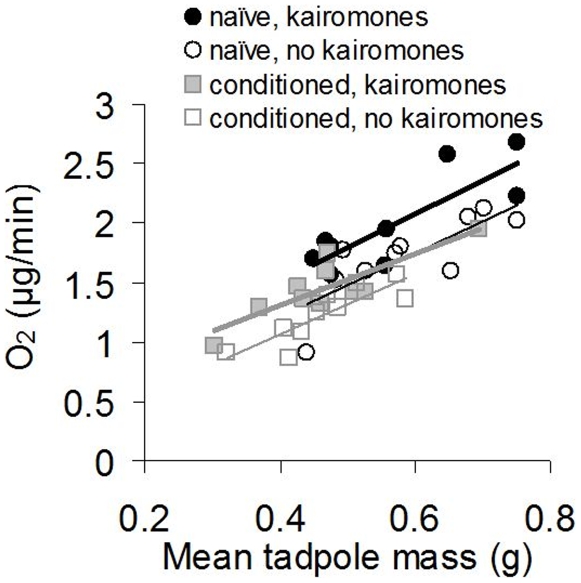
Relationship between oxygen consumption and tadpole mass for conditioned and naïve tadpoles measured in environments with and without kairomones. Each point is the average of three tadpoles measured during three 4-minute intervals.

Oxygen consumption, corrected for body mass, was reduced 10.0% in conditioned tadpoles (0.287±0.02 µg/min per 100 mg mass) compared to naïve tadpoles (0.319±0.01 µg/min per 100 mg mass; Fig. 1, Table 1). Oxygen consumption increased 16.8% when tadpoles were measured in kairomone water (water containing predator cues; 0.312±0.01 µg/min per 100 mg mass) compared to blank water (water lacking predator cues; 0.267±0.01 µg/min per 100 mg mass). Kairomones are chemical cues emitted by predators that have fed upon prey [30]. There was no interaction between rearing and measuring environments. Time of day did not influence oxygen consumption, but temperature had a significant positive effect (Table 1; increase by 0.0211±0.0059 µg/min per °C for 100 mg mass).

**Table 1:** Mixed-effects model testing for the influence of body mass, temperature, time of day, rearing environment, and measuring environment on oxygen consumption of tadpoles.

Source	Estimate±1 SE	Test statistic	*P*-value
*Fixed effects*			
Body mass	7.343±0.822	8.932	0.0001
Temperature	1.210±0.313	3.862	0.0005
Time of measurement	−5.974±3.278	−1.823	0.0778
Rearing environment	−3.030±0.697	−4.348	0.0001
Measuring environment	−2.838±0.745	−3.806	0.0006
Rearing* Measuring	1.082±0.623	1.739	0.0917
*Random effect*			
Rearing pool	1.354	5.134	0.0189

The model included the rearing pool as random factor. Estimates and test statistics are the coefficient and *t*-value for fixed effects, and the variance component and LR statistic for the random effect. Significance of fixed effects was judged from 10,000 Markov chain Monte Carlo samples drawn from the posterior distribution of the parameters in a Bayesian version of the model.

## Discussion

We found that physiological responses to predation risk were highly plastic, and depended on the time scale of exposure to risk. Tadpole oxygen consumption increased during short term exposure to predation risk but declined after long term exposure. Our interpretation of these results assumes that oxygen consumption is correlated with metabolic rate [31], [32]; metabolism reflects energetic demand, which in turn links to our interest in the growth/predation risk tradeoff. Although our study does not reveal the origin of this tradeoff, it adds to our understanding of its underlying physiological mechanisms.

Increased oxygen consumption by naïve tadpoles under short-term exposure to predators parallels similar findings in other organisms [20]–[23]. This reaction is interpreted as a component of the “fight-or-flight” response, in which release of stress hormones triggers (among other things) increased respiration and heart rate, redirection of energy to locomotory structures, and an enhanced ability to escape predators [19], [33]. Naïve tadpoles might be expected to show a particularly strong response to short-term predator exposure, because kairomones represented a novel threat to them. This was not observed. The change in oxygen consumption caused by short-term exposure was roughly the same for both kinds of tadpoles; that is, naïve tadpoles increased their oxygen consumption when faced with kairomones by about the same amount as conditioned tadpoles reduced oxygen consumption when suddenly released from predation risk. The physiological response to predation risk is therefore nearly instantaneous, which implies a rapid and accurate assessment of the chemical environment. This result also shows that oxygen consumption is not closely linked to behaviour, because tadpoles released from predation risk do not show an immediate change in feeding or swimming activity to match the novel predator-free environment [29], [34], [35].

Our discovery that tadpoles decreased oxygen consumption after long term exposure to predators is unexpected in light of the short-term response to kairomones. But this result is supported by other work showing that vertebrates can have distinct short-term and long-term physiological responses to stress. While metabolic rate typically increases under sudden exposure to stress [20], [22], [33], it can decline over long-term stress [36] or long-term implantation of stress hormones such as corticosterone [37]. Thus, the conditioned tadpoles in our study reacted as other vertebrates do when they experience extended exposure to stress hormones.

What are the consequences of these short- and long-term physiological responses for the growth/predation risk tradeoff? Over short time periods, there are potentially costly reactions at the physiological level (oxygen consumption is increased [20]–[23, this study]) and the behavioural level (feeding is curtailed [8], [38]). The physiological reaction diverts energy from growth or storage into metabolism [24]–[26] and the behavioural reaction affects food intake [7], [8]. But the impact of these events on individual growth rate will be small if the fight-or-flight response lasts for a relatively short time. Our study was not designed to detect the duration of the short-term metabolic response, but reversibility of various predator-induced responses suggests that the impact might not be long lasting [34]. Over the long-term, there is acclimation to predation risk at both physiological and behavioural levels, such that oxygen consumption declines (this study) and food intake rebounds to that observed in low-risk situations [3], [13]. Thus, allocation theory suggests that long-term changes in metabolism and food consumption cannot explain the growth costs of responding to predators found in this study. In fact, a plausible interpretation of our results is that the metabolic response has evolved to minimize costs of anti-predator defence. However, those costs that remain must originate elsewhere.

This conclusion may at first seem discouraging, but we prefer to emphasize that a physiological approach to inducible defences holds much promise for understanding the growth/predation risk tradeoff. For instance, the short term response observed here and in previous studies demonstrates a highly accurate and rapid assessment of the environment, enabling instantaneous and reversible plasticity. Recent studies of anurans and other taxa likewise illustrate complex interactions among predation risk, behaviour, metabolism, and enzyme physiology [13], [15], [19], [20, this study]. Many more induced physiological mechanisms surely await discovery.

## Materials and Methods

Tadpoles of *Rana temporaria* Linnaeus, 1758, react to predators by decreasing feeding and swimming activity and increasing the depth of their tail fins, both of which reduce vulnerability to predation [9], [39]. Predators also cause reductions in growth and development rates, which are usually construed as costs of defence [12], [29], [40]. We first reared tadpoles for three weeks with and without non-lethal predators (termed conditioned and naïve tadpoles), and then tested the oxygen consumption of both types of tadpole in the presence and absence of predator kairomones. Our experiment had a two-by-two factorial design, with long-term conditioning environment crossed with testing environment.

### Rearing of conditioned and naïve tadpoles

Tadpoles were reared outdoors in 20 plastic pools (0.28 m^2^, 80 litres volume), giving 10 replicates each of two treatments (with and without predators). The pools were filled with aged tap water, covered with shade cloth to prevent colonization by predators, and stocked with zooplankton, 5 g of rabbit food, and 60 g of dried leaf litter. We arranged pools in a field at the University of Zürich, Switzerland, and assigned treatments at random. The predator pools contained a floating cage (∼1 litre volume) containing one final instar dragonfly larva (*Aeshna cyanea* Müller, 1764). Throughout the rearing period the predators were fed 300 mg of *R. temporaria* tadpoles three times a week and were rotated to equalize any possible differences between individual dragonfly larvae. Pools without predators contained empty cages, which were also rotated to control for effects of disturbance. Tadpoles were derived from three clutches collected on the university campus; each pool received 15 (five from each clutch) randomly assigned, six day old tadpoles on 5 April 2004.

### Oxygen consumption

We measured oxygen consumption over three consecutive days (27–29 April 2004) using an intermittently closed respirometer in which a measuring period alternated with a flow-through period [41], [42]. The respirometer consisted of an aquarium pump, a sequencing valve system, a stirring chamber with a HQ20 LDO sensor (Hach-Lange GmbH, Hegnau, Switzerland), and two experimental chambers (each 125 ml volume), all immersed in a 120 L aquarium. We conducted 20 trials comprising 40 groups in all. Each trial included two groups of three tadpoles, each of which was randomly assigned to one of the two experimental chambers. In each trial one group originated from a rearing pool with predators (conditioned tadpoles), while the other group originated from a pool without predators (naïve tadpoles). Trials lasted for 30 minutes, during which 5-min intervals of flow-through were alternated with five minutes of measuring. While one chamber was measured the other was flushed. The oxygen sensor made recordings every 30 sec. Immediately after a chamber switched from flow-through to measurement, there was a brief period during which the remaining water in the hoses and stirring chamber mixed with the water from the measurement chamber. We therefore discarded data from the first minute of each measuring period. For logistical reasons we could not randomize the sequence of exposure to water with kairomones and blank water (without kairomones). Thus, we started each day with trials in blank water and thereafter added 200 ml of water containing kairomones (from three *A. cyanea* larvae each held in 200 ml water and fed 300 mg *R. temporaria* tadpoles two days earlier). We allowed the kairomones to mix for 15 minutes before initiating trials under kairomone conditions. Each evening the aquarium and respirometer were cleaned and refilled for the following day's trials.

After each trial the wet mass of both groups of three tadpoles was recorded. The temperature in the blank environment (18.17±0.20°C) was lower than that in the kairomone environment (19.39±0.17°C). We analyzed the data using a mixed effect model with average oxygen consumption across the three 4-min measuring periods (μg/min) as the response variable, rearing pool as a random factor, mass, temperature, time of measurement, and measuring environment, as fixed effects measured at the level of the group, and the rearing treatment as a fixed effect at the level of the pool. Time was included to account for changes in metabolic rate during the day [43], [44], because trials in blank water were performed prior to the trials in kairomone water. We judged the significance of fixed effects from 10,000 Markov chain Monte Carlo samples drawn from the posterior distribution of the parameters in a Bayesian version of the model [45]. Analyses were done using the lmer function in R [46]. One group of tadpoles was discarded because their experimental chamber opened prematurely.
